# Enrichment of RedoxifibromiR miR-21-5p in Plasma Exosomes of Hypertensive Patients with Renal Injury

**DOI:** 10.3390/ijms26020590

**Published:** 2025-01-12

**Authors:** Olga Martinez-Arroyo, Ana Flores-Chova, Marta Mendez-Debaets, Sergio Martinez-Hervas, Fernando Martinez, Maria J. Forner, Josep Redon, Ana Ortega, Raquel Cortes

**Affiliations:** 1 Cardiometabolic and Renal Risk Research Group, INCLIVA Biomedical Research Institute, 46010 Valencia, Spain; omartinez@incliva.es (O.M.-A.); aflores@incliva.es (A.F.-C.); mmendez@incliva.es (M.M.-D.); fernandoctor@hotmail.com (F.M.); maria.jose.forner@uv.es (M.J.F.); josep.redon@uv.es (J.R.); aortega@incliva.es (A.O.); 2 Cardiometabolic Group, INCLIVA Biomedical Research Institute, 46010 Valencia, Spain; sergio.martinez@uv.es; 3 Endocrinology and Nutrition Unit, Hospital Clinico Universitario, 46010 Valencia, Spain; 4 Department of Medicine, University of Valencia, 46010 Valencia, Spain; 5 Diabetes and Associated Metabolic Diseases (CIBERDEM), Institute of Health Carlos III, Minister of Science, Innovation and Universities, 28029 Madrid, Spain; 6 Internal Medicine Unit, Hospital Clinico Universitario, 46010 Valencia, Spain; 7 CIBER of Cardiovascular Diseases (CIBERCV), Institute of Health Carlos III, Minister of Science, Innovation and Universities, 28029 Madrid, Spain

**Keywords:** microRNA, fibromiR, redoximiR, exosomes, hypertension, renal damage, albuminuria

## Abstract

Several microRNAs (miRNAs) emerged as powerful regulators of fibrotic processes, “fibromiRs”, and can also influence the expression of genes responsible for the generation of reactive oxygen species, “redoximiRs”. We aimed to investigate whether plasma exosomes from hypertensive and diabetes patients are enriched in fibromiRs and redoximiRs using deep sequencing technology and their association with relevant signalling pathways implicated in oxidative stress and fibrogenesis by GO terms and KEGG pathways. RNA-Seq analysis from P-EXO identified 31 differentially expressed (DE) miRNAs in patients compared to controls, of which 77% are biofluid specific. The majority of the exosomal DE miRNAs were identified as fibromiRs (55%) or redoximiRs (26%). One of the most representative miRNAs identified was miR-21-5p, of which levels in P-EXO were increased by 3.83-fold change (*p* < 0.0001) in hypertensive patients with albuminuria and were highly associated (r Spearman = 0.64, *p* < 0.0001). In addition, P-EXO miR-21-5p had a high accuracy in discriminating renal damage (AUC = 0.82, *p* < 0.0001). Bioinformatic analysis revealed that miR-21-5p regulates key pathways in the context of organ fibrosis, such as chemokine, Ras, and MAPK signalling. Additionally, in vitro studies showed an increase in P-EXO miR-21-5p levels after TGF-β1 damage and oxidative stress. This novel study found an enrichment of fibromiRs and redoximiRs in P-EXO from hypertensive/diabetic patients with renal dysfunction. miR-21-5p, such as a RedoxifibromiR, has a significant accuracy for discriminating renal damage and is closely related with relevant signalling pathways implicated in fibrogenesis in podocytes.

## 1. Introduction

Hypertension (HTN) is a prevalent chronic condition, which together with type 2 diabetes, contributes significantly to the global burden of renal dysfunction [1]. As a major risk factor for the development and progression of chronic kidney disease (CKD), HTN leads to substantial increases in morbidity and mortality [2]. The pathophysiology of renal dysfunction in hypertensive patients is complex, involving various molecular and cellular mechanisms [3]. Among these mechanisms, oxidative stress and fibrosis are particularly important, as they play key roles in the progressive deterioration of renal function [4]. Oxidative stress in the kidneys results in the overproduction of reactive oxygen species (ROS), which damage renal cells and tissues, thereby triggering inflammatory responses and fibrotic processes. Fibrosis, characterized by the excessive accumulation of extracellular matrix proteins, contributes to the scarring and stiffening of the kidney tissue, further impairing renal function.

The term extracellular vesicle (EV) comprises a heterogeneous population of cell-derived membranous structures, including exosomes, microvesicles (MVs), and apoptotic bodies that can be established based on their size and biogenesis [5,6]. Exosomes, small extracellular vesicles ranging from 30 to 150 nm in diameter, emerged as critical mediators of intercellular communication [7]. They carry a diverse array of bioactive molecules, including proteins, lipids, and nucleic acids such as microRNAs (miRNAs) [8]. miRNAs are small, non-coding RNAs that regulate gene expression post-transcriptionally and are implicated in numerous physiological and pathological processes [9]. Specific subsets of miRNAs, termed RedoximiRs and FibromiRs, were identified to regulate oxidative stress and fibrogenesis, respectively [10,11,12].

Recent studies, including studies from our group, highlighted the potential of exosomal miRNAs as biomarkers for various diseases, including HTN and renal dysfunction in lupus [13,14,15]. Exosomal miRNAs are stable in circulation, reflect the pathological state of their cells of origin, and can be detected non-invasively in body fluids such as blood and urine [16,17]. This makes them attractive candidates for diagnostic and prognostic markers, as well as for therapeutic targets. Therefore, investigating the role of exosomal miRNAs in renal dysfunction could provide valuable insights into disease mechanisms and lead to the development of non-invasive biomarkers for early diagnosis and monitoring of therapeutic response.

In this study, we investigate the miRNA profile of Plasma EXOsomes (P-EXO) derived from hypertensive patients with and without albuminuria. We focus on the enrichment of specific RedoximiRs and FibromiRs and their association with relevant signalling pathways implicated in oxidative stress and fibrogenesis. Then, we analyse the behaviour of the miRNA identified in an in vitro model of a key glomerular cell, podocytes, under pro-fibrotic and oxidative stress conditions.

## 2. Results

### 2.1. Study Cohorts

The discovery cohort was formed by 15 hypertensive patients with DN and healthy control n = 25 (Supplemental Table S1). The validation population included 66 essential HTN subjects, 40 subjects with increased Urinary Albumin Excretion (UAE), and 26 non-UAE. General patient characteristics and are shown in Table 1. Hypertensive patients with UAE were older and had higher blood pressures, triglycerides, plasma creatinine, Glomerular Filtration Rate (GFR), and albuminuria than control (CNT) group. In addition, the incidence of diabetes mellitus, obesity, hyperlipidaemia, and smoking was augmented in the UAE group.

### 2.2. Differential Expression of miRNAs in Plasma and P-EXO

**Figure 1:**
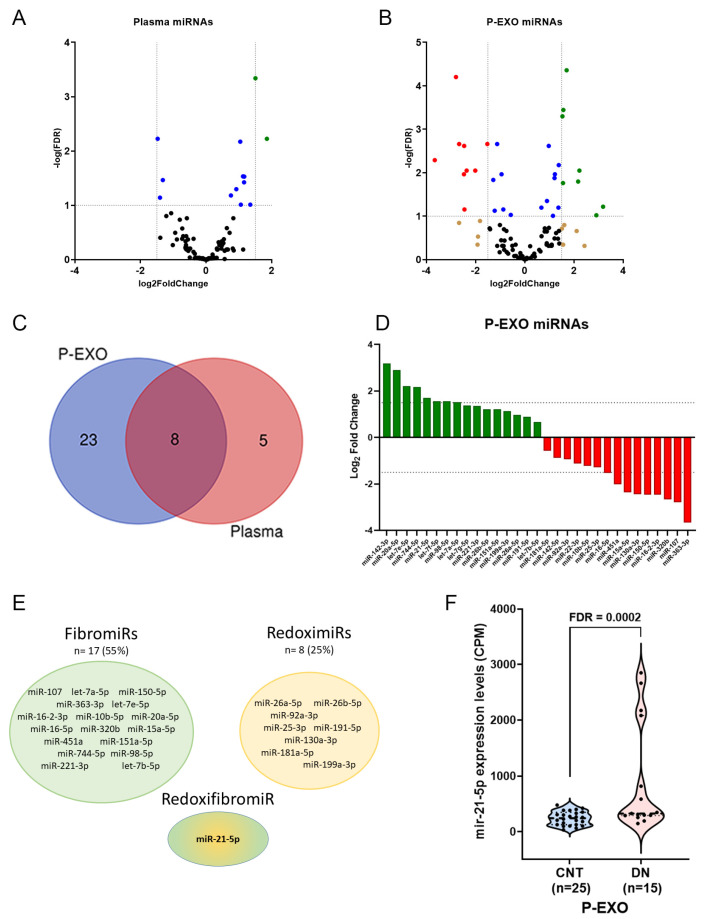
Differentially expressed (DE) miRNAs in plasma and P-EXO of CNT versus DN patients adjusted by age and gender. (**A**) Volcano plot depicts significantly altered plasma miRNAs and (**B**) plasma exosomes in diabetic nephropathy (DN). Each dot represents an RNA; non-significant false discovery rate (FDR > 0.05) and log2 fold change ≥ −1.5 or ≤1.5) in black, with log2 fold change ≤ −1.5 or ≥1.5 in brown, with significant FDR in blue and with significant FDR and log2 fold change ≥ 1.5, green (upregulated) or ≤−1.5 in red (downregulated). (**C**) Venn diagram shows the overlap among biological fractions. (**D**) Diverging bar charts give the fold change expression of the exosomal miRNAs signature in DN: upregulated are in green and downregulated in red. (**E**) Schematic image for the FibromiRs and RedoximiRs in P-Exo from DN and the RedoxifibromiR miR-21-5p. (**F**) Violin plot of P-Exo miR-21-5p levels between controls (CNT) and DN. Data are counts per million (CPM) from RNA Seq analysis.

RNA-Seq analysis was conducted to identify Differentially Expressed (DE) miRNAs in plasma and P-EXO of CNT versus DN patients adjusted by age and gender. As shown in the volcano plot (Figure 1A,B), the analysis of miRNA in all patients for each biofluid identified more significant RNAs differentially expressed (DE) (FDR < 0.05) in P-EXO fraction than in plasma. Notably, the miRNA profiles differed between plasma and P-EXO fractions. Out of the 31 DE miRNAs identified, 23 were specific to P-EXO, 5 were specific to plasma, and 8 miRNAs were common to both biofluids. This indicates that 74% of the P-EXO miRNAs were biofluid-specific, highlighting the distinct miRNA profiles between exosomal and plasma fractions (Figure 1C). Figure 1D illustrates the log2 fold change in the levels of DE P-EXO miRNAs between CNT and DN groups. Approximately half of the DE miRNAs were significantly upregulated in DN patients, while the other half were markedly downregulated. Among the DE miRNAs, a significant proportion were identified as FibromiRs (17 miRNAs, 55%), which are known to be involved in fibrosis, and RedoximiRs (8 miRNAs, 25%), which are implicated in oxidative stress pathways (Figure 1E). In particular, miR-21-5p was highlighted due to its known involvement in both fibrotic and oxidative pathways, as indicated in previous analyses [10]. Interestingly, miR-21-5p was significantly upregulated in P-EXO from DN samples (FDR = 0.0002), as shown in the violin plot (Figure 1F). This suggests that miR-21-5p may serve as a robust biomarker for disease detection and progression.

### 2.3. Elevated miR-21-5p Levels in P-EXO of Hypertensive and Albuminuric Patients

**Figure 2:**
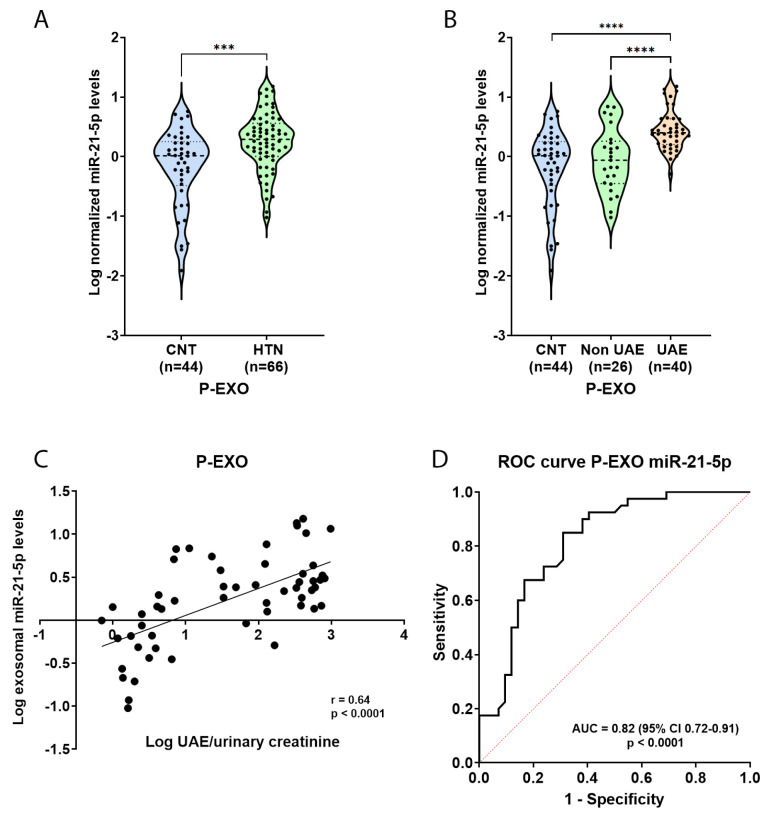
Validated miR-21-5p levels in P-EXO and association with albuminuria in hypertensive patients. (**A**) Violin plot graph of P-Exo miR-21-5p levels comparison between controls (CNT) and hypertensive patients (HTN). (**B**) Violin plot graph of P-Exo miR-21-5p levels comparison between HTN with albuminuria (HTN UAE), without (HTN Non-UAE), and CNT. (**C**) Association between P-EXO miR-21-5p and log UAE/urinary creatinine in HTN patients. Spearmen correlation analysis was employed. (**D**) P-Exo miR-21-5p receiver operating characteristic curve for the detection of albuminuria. AUC, area under the curve. *** *p* < 0.001; **** *p* < 0.0001.

To validate the findings from the discovery cohort, we examined miR-21-5p levels in a cohort of hypertensive patients by Real-Time quantitative PCR (RT-qPCR). The validation cohort included CNT, n = 44, and HTN n = 66. Analysis of miR-21-5p expression in P-EXO revealed a significant increase in HTN patients compared to CNT (*p* < 0.001, Figure 2A). When stratifying the hypertensive group based on UAE, miR-21-5p levels were significantly elevated in albuminuric hypertensive patients (HTN UAE group) compared to both controls and non-albuminuric hypertensive patients (HTN Non-UAE group) (*p* < 0.0001, Figure 2B).

Further analysis was conducted to explore the relationship between miR-21-5p levels in P-EXO and UAE. A strong positive correlation was observed between miR-21-5p levels in P-EXO and the urinary albumin-to-creatinine ratio (UAE/creatinine) (r = 0.64, *p* < 0.0001, Figure 2C). Then, when we performed a multiple linear regression adjusted by age, gender, blood pressures, Body Mass Index (BMI), presence of diabetes, dyslipidaemia, and smoking, P-EXO miR-21-5p was an independent predictor associated with log UAE/U-creatinine (*p* < 0.0001), suggesting that miR-21-5p levels in P-EXO reflect the severity of albuminuria.

Finally, to evaluate the diagnostic value of miR-21-5p in discriminating HTN albuminuric patients (HTN UAE group) from CNT, a receiver operating characteristic (ROC) curve was generated. The Area Under the Curve (AUC) was 0.82 [95% Confidence Interval (CI): 0.72–0.91, *p* < 0.0001], indicating good discriminatory power (Figure 2D). These results reinforce the potential of P-EXO miR-21-5p as a non-invasive biomarker of kidney damage in HTN.

### 2.4. Identification of miR-21-5p Targets and Functional Enrichment Analysis

**Figure 3:**
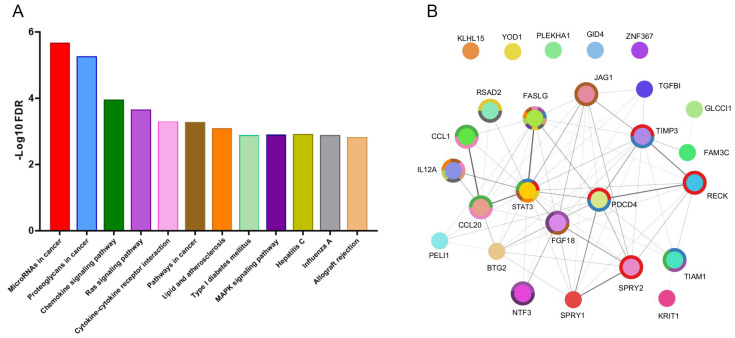
Regulatory network of miR-21-5p for exosomal fraction in albuminuria. Pathway enrichment analysis using KEGG database (**A**); their predicted targeted genes (colour circles) (**B**), joint to the top 12 KEGG pathways with a donut colour that is associated to each target.

Using an integrative approach involving three web-based tools—TargetScan, miRDB, and miRTarBase—we identified multiple targets of miR-21-5p. The resulting network diagram (Figure 3) illustrates the complex interactions among these targets. Central nodes in the network, such as STAT3, FASLG, and FGF18, suggest a high degree of connectivity, implying their significant role in the regulatory network governed by miR-21-5p. Key nodes like STAT3, FASLG, TIMP3, and PDCD4 exhibit extensive interactions, indicating their potential critical roles in the miR-21-5p regulatory mechanism.

Pathway enrichment analysis using Kyoto Encyclopedia of Genes and Genome (KEGG database revealed several pathways significantly regulated by miR-21-5p, as depicted in the bar chart (Figure 3). Bioinformatic analysis revealed that miR-21-5p, a pro-fibrotic redoximiR, regulates essential signalling pathways pertinent to fibrosis, including the chemokine, Ras, and Mitogen-Activated Protein Kinase (MAPK) pathways. These findings highlight the central role of miR-21-5p in fibrosis-related cellular processes and signalling mechanisms. The enrichment ratios, gene counts, and FDR for each 12 KEGG pathways are shown in Supplemental Table S2.

### 2.5. Functional In Vitro Experiments

**Figure 4:**
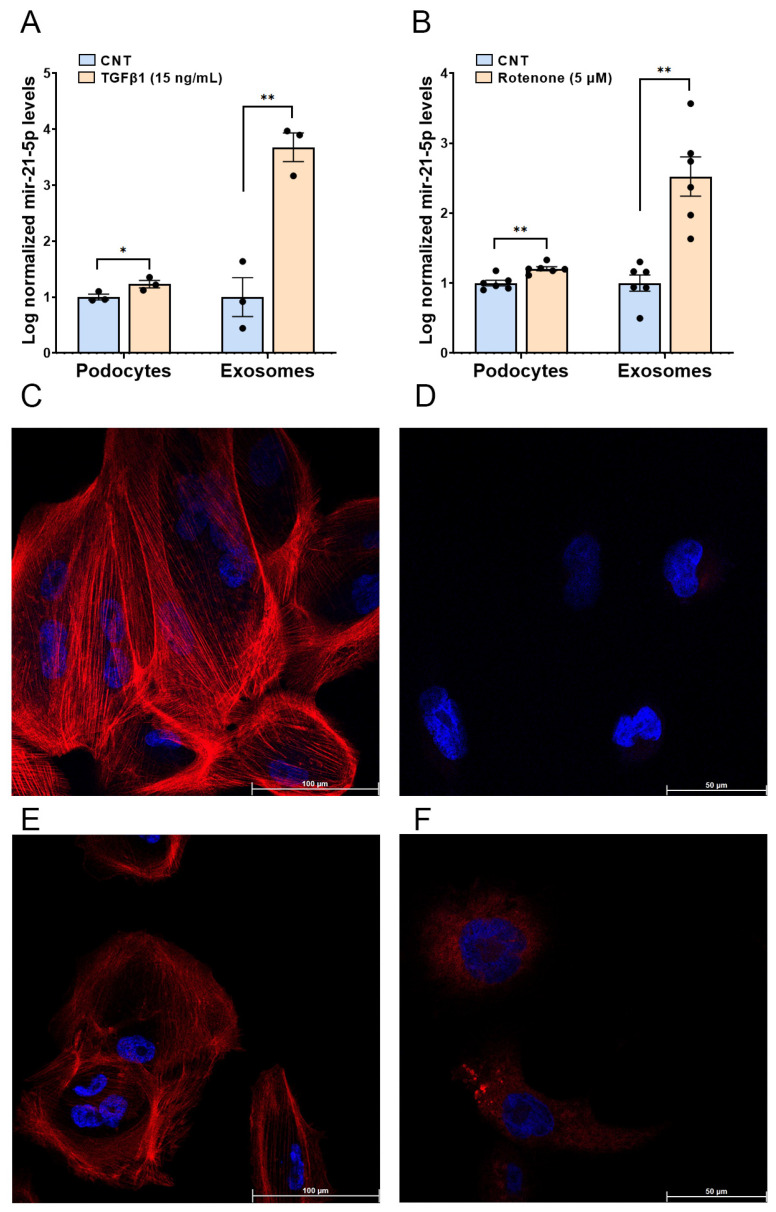
Effect of TGF-β1 and rotenone treatments on miR-21-5p in human podocytes. (**A**) Expression levels of miR-21-5p after stimulation with TGF-β1 in podocytes and their exosomes, and the effect on F-actin architecture (**C**) control and TGF-β1 (15 ng/mL) (**E**) by confocal microscopy. (**B**) Expression levels of miR-21-5p after treatment with rotenone (5 µM), and the effect on superoxide production in human podocytes (**D**–**F**) by confocal microscopy. * *p* < 0.05; ** *p* < 0.01.

As Tumour Growth Factor-β1 (TGF-β1) signalling was a biological process regulated by the differentially expressed miR-21-5p in our patients, we analysed the impact of TGF-β1, the major factor driving renal fibrosis in podocytes and podocyte-derived exosomes [18,19]. Figure 4A illustrates the effect of TGF-β1 (15 ng/mL) on miR-21-5p levels, in podocytes. miR-21-5p levels show a modest increase (20%, *p* < 0.05). In contrast, exosomal miR-21-5p levels increase significantly (3.7-fold increase, *p* < 0.01). Regarding oxidative stress, Figure 4B. shows that rotenone (5 µM), also significantly increases miR-21-5p levels in both podocytes (20%, *p* < 0.01) and even more elevated in their derived exosomes (2.52-fold change, *p* < 0.01), suggesting that rotenone may promote both the production and exosomal export of miR-21-5p.

To ensure that in vitro treatments exerted the expected profibrotic and oxidative stress effects, we analysed the distribution of the F-actin cytoskeleton, involved in maintaining podocyte cytoarchitecture using immunofluorescence. Additionally, we confirmed oxidative stress induction by the applied reagent, rotenone, by measuring superoxide production with the MitoSOX probe (Figure 4C,D, respectively). Both treatments produce podocyte alterations, in F-actin cytoarchitecture and increase in oxidative stress (Figure 4E,F).

## 3. Discussion

Our study demonstrated a significant enrichment of specific redox- and fibrosis-related miRNAs in plasma exosomes from hypertensive patients with renal dysfunction, highlighting miR-21-5p. This enrichment underscores the role of exosomal miR-21-5p as a biomarker and potential mediator of renal damage in the context of HTN. Previous studies highlighted miR-21-5p as a key factor in fibrosis and oxidative stress pathways, which are central to the pathophysiology of HTN0derived renal damage [12,20,21]. Consistent with these findings, our data showed that hypertensive patients with increased UAE presented elevated levels of exosomal miR-21-5p associated to UAE values and its power for discriminating renal damage presence indicating its potential as a non-invasive biomarker for renal injury severity.

The observed upregulation of miR-21-5p in P-EXO is consistent with the established role of miRNAs in exosomes as mediators of intercellular signalling in several disease contexts [13,14,22]. miR-21-5p, in particular, was implicated in the regulation of cytokine activities, which further contributes to the inflammatory and fibrotic environment in CKD [23]. Our study builds on these findings by showing that hypertensive patients exhibit increased levels of exosomal miR-21-5p in correlation with UAE.

Previous analysis revealed that miR-21-5p is connected to signalling pathways such as STAT3 and Interleukin-12 (IL-12), both crucial in inflammation and fibrosis processes [24,25]. The network analysis highlights miR-21-5p’s regulatory potential, suggesting it could serve as a therapeutic target. Moreover, miR-21-5p’s ability to interact with multiple target pathways in fibrotic signalling enhances its candidacy as a therapeutic focus [26]. For example, FasLG-Fas system regulates renal cell apoptosis, as well as the immune and inflammatory responses participating in renal injury [27]. Its role in the regulation of extracellular matrix production, combined with the upregulation observed in hypertensive renal dysfunction, suggests that the targeted modulation of miR-21-5p could reduce fibrosis progression in renal damage. Our findings aligned with the literature, showing miR-21-5p’s involvement in key signalling pathways related to fibrosis, including MAPK and TGF-β signalling, through STAT3 that selectively interacts with Smad3 to antagonize TGF-β promoting fibrosis [28].

The correlation between miR-21-5p and fibrosis markers, particularly through the TGF-β1 pathway, is well documented, where miR-21-5p was shown to exacerbate fibrosis through TGF-β1 signalling [29,30,31]. Podocytes are critical cells in the kidney that maintain the filtration barrier, and their dysfunction plays a central role in the progression of renal disease. In our study, in vitro experiments further demonstrated that stimulation with TGF-β1 upregulated exosomal miR-21-5p levels in podocytes more significantly than intracellular miR-21-5p levels. This suggests that TGF-β1 may influence renal fibrosis partly via exosome-mediated signalling, with miR-21-5p acting as a potent fibrosis mediator in renal damage. These results align with findings from other studies that identify exosomal miR-21-5p as a contributor to renal fibrosis through extracellular matrix production and podocyte injury [21,32]. However, more in-depth functional studies are required to support this finding.

Another significant result in our study is the upregulation of exosomal miR-21-5p upon rotenone-induced oxidative stress. Mitochondrial dysfunction, often linked to increased ROS production, was implicated in the activation and release of fibrosis-associated miRNAs like miR-21-5p [33,34]. In our in vitro model, rotenone treatment, a mitochondrial inhibitor, increased miR-21-5p levels in podocytes and their derived exosomes, suggesting that cells under oxidative stress may release higher levels of miR-21-5p as a response to mitochondrial impairment. This aligns with other research demonstrating that miR-21-5p upregulation in response to oxidative stress contributes to renal fibrosis and exacerbates cellular damage by targeting genes involved in extracellular matrix (ECM) degradation, such as Metalloprotease-9 (MMP9) and Metallopeptidase Inhibitor 3 (TIMP3) [35]. Additionally, we noted a more pronounced increase in miR-21-5p levels within exosomes compared to podocytes when exposed to both TGF-β1 and rotenone. This suggests that podocytes may actively package and release miR-21-5p into exosomes, potentially enabling the transmission of fibrotic signals to other cells. Several factors could contribute to this process. First, TGF-β1 was shown to promote exosome production, which may account for the increased miR-21-5p levels in exosomes [36,37,38]. Additionally, under fibrotic stress, podocytes might selectively sort miR-21-5p into exosomes as a regulatory mechanism to modulate the fibrotic environment. The potential of exosomal miR-21-5p as a biomarker is further supported by studies linking its levels to disease severity and progression in kidney and liver fibrosis [29,39]. Exosomal miR-21-5p’s stability in circulation and its association with fibrosis make it a valuable candidate for assessing disease progression and therapeutic response.

These results indicate that P-EXO miRNAs provide a distinct and biologically relevant miRNA signature compared to plasma. The identification of FibromiRs, RedoximiRs, and the overlapping RedoxifibromiR miR-21-5p underscores the potential of P-EXO miRNAs in elucidating disease mechanisms and serving as non-invasive biomarkers. By elucidating the role of plasma exosomal miRNAs in these processes, we hope to pave the way for novel diagnostic and therapeutic strategies to combat hypertension and its related complications in this high-risk population. Identifying and targeting specific exosomal miRNAs could lead to the development of innovative treatments that mitigate renal damage and improve outcomes for hypertensive patients with kidney injury. Future studies should explore the therapeutic inhibition of miR-21-5p, particularly within the TGF-β1 signalling axis, as a means of attenuating renal fibrosis, so knockdown and overexpression experiments should be performed. Further validation in larger patient cohorts and longitudinal studies will be essential to establish exosomal miR-21-5p as a reliable clinical biomarker for hypertensive derived renal damage severity and progression. Exploring delivery mechanisms and evaluating the safety of miRNA-based therapies will be crucial for clinical application.

## 4. Materials and Methods

### 4.1. Patients Cohort

The discovery cohort was formed by patients with diabetic nephropathy (DN), all of them with HTN (n = 15) and healthy subjects as controls (CNT) (n = 25), we analysed the miRNA profile in P-EXO by next-generation sequencing (RNA-Seq). The validation population included essential hypertensive subjects (n = 66), 40 subjects with increased urinary albumin excretion (HTN UAE) and 26 with normoalbuminuria levels (HTN Non-UAE). Proteinuria was assessed in first voiding urine in the morning and expressed as the ratio with urinary creatinine (mg/g). UAE > 30 mg/g was considered albuminuria [40]. Patient recruitment was performed in collaboration with the Internal Medicine and Endocrinology and Nutrition Units of Hospital Clínico Universitario of Valencia (Spain).

### 4.2. Extracellular Vesicle (EV) Isolation from Plasma Samples and Podocyte Cell Cultures

Exosomes were isolated from plasma (P-EXO) using a differential ultracentrifugation protocol previously described by our group [14]. First, blood EDTA tubes and cell culture medium were centrifuged at 2250× *g* for 15 min to remove cells and debris. The resulting cell-free supernatant was then centrifuged at 20,000× *g* for 45 min (Ultracentrifuge Optima L 100K, 70 Ti rotor, Beckman Instruments, Brea, CA, USA) to separate larger EVs. Finally, the supernatant from this step was centrifuged at 110,000× *g* to collect the P-EXO-enriched pellet, which was resuspended in 0.2 µm filtered PBS and processed for downstream analyses.

### 4.3. RNA Extraction, Small RNA Library Preparation, and Next-Generation Sequencing

Total RNA was extracted from exosomes using the Total Exosome RNA and Protein Isolation kit (Invitrogen, Life Technologies, Carlsbad, CA, USA). The extracted RNA’s quantity, quality, and size distribution were assessed via capillary electrophoresis using an Agilent 2100 Bioanalyzer (Agilent Technologies, Santa Clara, CA, USA) with an RNA 6000 Pico chip.

Single-patient libraries were generated utilizing the Small RNA-Seq Library Prep Kit (Lexogen GmbH, Vienna, Austria) according to a protocol tailored for small RNA library preparation from low-input samples. In summary, adapter ligation and cDNA amplification were conducted, followed by size selection using the Pippin Prep Automated DNA Size Selection System (Sage Science, Beverly, MA, USA). Libraries were then purified, concentrated, and their quality was assessed through capillary electrophoresis on the QIAxcel Advanced System (Qiagen, Hilden, Germany) before quantification via real-time quantitative PCR (RT-qPCR). Sequencing was performed on the NextSeq 550 platform (Illumina, San Diego, CA, USA) using paired-end reads with a 2 × 150-cycle configuration.

In summary, for small-RNA sequencing (RNA-seq), the featureCounts function available in Bioconductor R package Rsubread was used to extract the normalized read count from the Small RNA-Seq data, after mapping the trimmed reads by Trim Galore!, using STAR against the last version of the human reference genome (GRCh38). The mature transcripts of miRNAs were obtained from miRBase using the featureCounts function. The differential expression levels of miRNAs were calculated with the Bioconductor DESeq2 package for the R software (v. 4), which allows us to adjust the analysis by age and gender, including these additional covariates in the design of the formula [15]. The raw RNA-Seq dataset is available at the BioProject repository, accession: PRJNA1189327.

### 4.4. miRNA Levels Quantification by RT-qPCR

To validate the RNA-seq data, we performed RT-qPCR analysis on the selected exosomal miRNA, miR-21-5p, using TaqMan™ Advanced miRNA Assays (Applied Biosystems, MA, USA). The TaqMan™ Advanced miRNA cDNA Synthesis Kit was used to synthesize cDNA from 2 μL of total RNA extracted from each sample. The measurements were carried out using the LightCycler^®^ 480 II real-time PCR system (Roche diagnostics, Barcelona, Spain). The miRNAs were chosen based on their presence in 100% of samples in both study groups, an absolute log2 fold change (FC) of ≥1.5 or ≤−1.5 and a False Discovery Rate (FDR) < 0.05, and a base mean > 10, from next-generation sequencing data. The absolute copy number for miR-21-5p was calculated using a standard curve prepared with a synthetic miRNA and two miRNAs were selected as internal controls for our experiment (miR-125a-3p and miR-186-5p) due to their stable expression across samples and fractions. miRNA values were expressed as the log ratio between the miRNA values and the mean value of internal control. For RT-qPCR studies performed on in vitro cultures, the synthetic miR-cel39-3p and ath-miR-159a were used for data normalisation.

### 4.5. Molecular Pathways Analyses

To identify miRNA targets, we utilized three web-based tools: TargetScan, miRDB, and miRTarBase. Targets were selected based on the following criteria: for TargetScan, a cumulative weighted context++ score of <−0.5; for miRTarBase, targets supported by more than one research paper or validated by multiple methods; and for miRDB, a Target Score of 90 or higher [15]. Only targets predicted by at least two of these tools were selected for further analysis. The URLs for these tools are TargetScan: http://www.targetscan.org/vert_72/ (accessed on 11 October 2024), miRDB: http://mirdb.org/ (accessed on 11 October 2024), and miRTarBase: https://mirtarbase.cuhk.edu.cn/∼miRTarBase/miRTarBase_2025 (accessed on 11 October 2024). Additionally, Gene set Over-Representation Analysis (ORA) was performed through GO terms and KEGG pathways in WebGestalt [Gene SeT AnaLysis Toolkit (http://www.webgestalt.org/ (accessed on 10 November 2024)). Protein–protein interactions were also accessed through the STRING v11.5 software.

### 4.6. Podocyte Cell Culture and Stimulation with TGF-β1 and Rotenone

Human immortalized podocytes were cultured and differentiated for 10 days in Roswell Park Memorial Institute 1640 (RPMI 1640) medium. Following differentiation days, podocytes were cultured in RPMI 1640 with 0% Foetal Bovine Serum (FBS) for 24 h [41]. Subsequently, the podocytes were stimulated with recombinant human TGF-β1 at concentrations of 15 ng/mL and rotenone at 5 µM for up to 24 h, both reagent from Sigma-Aldrich, St. Louis, MI, USA. Finally, changes in miR-21-5p levels in both exosomal and cellular fractions after the TGF-β1 and rotenone stimulation were measured by RT-qPCR.

### 4.7. Immunofluorescence Analysis

Immunofluorescence experiments were conducted as described in [41]. In brief, samples were fixed with 4% paraformaldehyde (PFA), permeabilized, and blocked with PBS containing 1% BSA. For visualizing the F-actin cytoskeleton in treated podocytes, cells were incubated with phalloidin-iFluor 594 (1:1000, Abcam, Cambridge, UK) for 1 h at room temperature, with DAPI added in the final 30 min. After incubation, cells were washed, mounted, and imaged. To assess oxidative stress in the in vitro model, MitoSOX Red staining (Thermo Fisher Scientific, Waltham, MA, USA) was used to detect mitochondrial superoxide production. Podocytes cultured on glass coverslips were treated with rotenone and then stained with 1 µM MitoSOX Red in HBSS for 30 min at 37 °C. Cells were subsequently rinsed in PBS, fixed with 4% PFA for 10 min. After washing, samples were mounted using ProLong™ Gold mounting medium (Thermo Fisher Scientific). Fluorescence was observed with a Leica TCS-SP8 confocal laser scanning microscope (Leica Microsystems, Wetzlar, Germany).

### 4.8. Statistical Analysis

Statistical analyses were conducted using GraphPad Prism (v.9.0) and SPSS software (v.20). The normality of each variable was assessed with the Kolmogorov–Smirnov test. Clinical variables were reported as mean ± SD for continuous variables or as percentages for categorical variables. Comparisons between groups for the categorical variables were performed with the χ2 test and the Student *t* test for continuous variables. Mann–Whitney U test was used to determine significant differences in exosomal miRNA levels among patient groups and for human podocyte experiments. Finally, Spearman correlation coefficient was used to determine the association between exosomal miRNA levels and UAE, and multiple lineal regression was performed for predicting the value of UAE based on P-EXO miR-21-5p levels along with more independent variables. The sensitivity and specificity of P-EXO miR-21-5p levels to discriminate the presence of renal damage was assessed by receiver operating characteristic (ROC) curves. A *p* < 0.05 was considered statistically significant.

## Figures and Tables

**Table 1:** Clinical characteristics of validation cohort.

Variables	HTN UAE (n = 40)	HTN NON-UAE (n = 26)	CNT (n = 44)
Age (years)	58 ± 11 ***	55 ± 6	41 ± 10
Gender (male)	74%	64%	57.0%
SBP (mmHg)	138± 19 ***	136 ± 24	117 ± 13
DBP (mmHg)	83 ± 11 ***	87 ± 14	73 ± 7
PP (mmHg)	54 ± 15 **	48 ± 17	43 ± 9
Glucose (mg/dL)	130 ± 50 **	119 ± 41	98 ± 12
Glycated hemoglobin (%)	6.9 ± 1.2 *	6.0 ± 0.9	5.6 ± 0.4
Total Cholesterol (mg/dL)	188 ± 35	173 ± 29	193 ± 41
LDL (mg/dL)	117 ± 31	108 ± 25	123 ± 34
HDL (mg/dL)	47 ± 13 **	50 ± 10	59 ± 14
Triglycerides (mg/dL)	188 ± 154 ***	127 ± 60	99 ± 87
Plasma creatinine (mg/dL)	1.01 ± 0.40 **	0.90 ± 0.22	0.76 ± 0.21
GFR (mL/min/1.73 m^2^)	86 ± 30 **	87 ± 19	107 ± 20
Body mass index (kg/m^2^)	33 ± 7 ***	30 ± 5	24 ± 4
Obesity grade (%)			
Grade I	21 **	18	2
Grade II	23 **	11	0
Grade III	13 **	11	0
Diabetes (%)	62 ***	64	2
Dyslipidemia (%)	90 ***	86	5
Smoking (%)	36 **	46	5
UAE/Creatinine (mg/g)	307 ± 231 ***	3.1 ± 1.7	4.4 ± 2.3
Treatment (%)			
Oral antidiabetic	54 **	32	2
CCB	39 **	38	5
ARA II	90 ***	93	5
Statins	32 **	8	5
BB	15.4 *	31	0
Diuretics	44 **	29	0

ARA II: Angiotensin Receptor Antagonists II; BB: Beta Blockers; CCB: Calcium Channel Blockers; CNT: control; DBP: Diastolic Blood Pressure; DN: Diabetic Nephropathy; GFR: Glomerular Filtration Rate; HDL: High-Density Lipoprotein; HTN: hypertension; LDL: Low-Density Lipoprotein; PP: Pulse Pressure; SBP: Systolic Blood Pressure; UAE: Urinary Albumin Excretion. Glomerular filtration rate (eGFR) was calculated from creatinine, age and sex using the CKD-EPI formula. * *p*-value <0.05; ** *p*-value < 0.01; *** *p*-value < 0.0001 CNT vs. MALB.

## Data Availability

The raw RNA-Seq dataset is available at the BioProject repository, accession: PRJNA1189327.

## Supplementary Materials

The following supporting information can be downloaded at: https://www.mdpi.com/article/10.3390/ijms26020590/s1.

## References

[1] Ohishi M. (2018). Hypertension with Diabetes Mellitus: Physiology and Pathology. Hypertens. Res..

[2] Olsen M.H., Angell S.Y., Asma S., Boutouyrie P., Burger D., Chirinos J.A., Damasceno A., Delles C., Gimenez-Roqueplo A.-P., Hering D. (2016). A Call to Action and a Lifecourse Strategy to Address the Global Burden of Raised Blood Pressure on Current and Future Generations: The Lancet Commission on Hypertension. Lancet.

[3] Burnier M., Damianaki A. (2023). Hypertension as Cardiovascular Risk Factor in Chronic Kidney Disease. Circ. Res..

[4] Touyz R.M., Rios F.J., Alves-Lopes R., Neves K.B., Camargo L.L., Montezano A.C. (2020). Oxidative Stress: A Unifying Paradigm in Hypertension. Can. J. Cardiol..

[5] György B., Szabó T.G., Pásztói M., Pál Z., Misják P., Aradi B., László V., Pállinger E., Pap E., Kittel A. (2011). Membrane Vesicles, Current State-of-the-Art: Emerging Role of Extracellular Vesicles. Cell. Mol. Life Sci..

[6] Colombo M., Raposo G., Théry C. (2014). Biogenesis, Secretion, and Intercellular Interactions of Exosomes and Other Extracellular Vesicles. Annu. Rev. Cell Dev. Biol..

[7] Martinez-Arroyo O., Ortega A., Redon J., Cortes R. (2021). Therapeutic Potential of Extracellular Vesicles in Hypertension-Associated Kidney Disease. Hypertension.

[8] O’Brien K., Breyne K., Ughetto S., Laurent L.C., Breakefield X.O. (2020). RNA Delivery by Extracellular Vesicles in Mammalian Cells and Its Applications. Nat. Rev. Mol. Cell Biol..

[9] Liu Y., Dong Z.-J., Song J.-W., Liang L.-R., Sun L.-L., Liu X.-Y., Miao R., Xu Y.-L., Li X.-T., Zhang M.-W. (2022). MicroRNA-122-5p Promotes Renal Fibrosis and Injury in Spontaneously Hypertensive Rats by Targeting FOXO3. Exp. Cell Res..

[10] Fierro-Fernández M., Miguel V., Lamas S. (2016). Role of redoximiRs in Fibrogenesis. Redox Biol..

[11] Pérez-Carrillo L., Giménez-Escamilla I., García-Manzanares M., Triviño J.C., Feijóo-Bandín S., Aragón-Herrera A., Lago F., Martínez-Dolz L., Portolés M., Tarazón E. (2023). Altered MicroRNA Maturation in Ischemic Hearts: Implication of Hypoxia on XPO5 and DICER1 Dysregulation and RedoximiR State. Antioxidants.

[12] Hennino M.-F., Buob D., Van der Hauwaert C., Gnemmi V., Jomaa Z., Pottier N., Savary G., Drumez E., Noël C., Cauffiez C. (2016). miR-21-5p Renal Expression Is Associated with Fibrosis and Renal Survival in Patients with IgA Nephropathy. Sci. Rep..

[13] Perez-Hernandez J., Riffo-Campos A.L., Ortega A., Martinez-Arroyo O., Perez-Gil D., Olivares D., Solaz E., Martinez F., Martínez-Hervás S., Chaves F.J. (2021). Urinary- and Plasma-Derived Exosomes Reveal a Distinct MicroRNA Signature Associated with Albuminuria in Hypertension. Hypertension.

[14] Riffo-Campos A.L., Perez-Hernandez J., Ortega A., Martinez-Arroyo O., Flores-Chova A., Redon J., Cortes R. (2022). Exosomal and Plasma Non-Coding RNA Signature Associated with Urinary Albumin Excretion in Hypertension. Int. J. Mol. Sci..

[15] Flores-Chova A., Martinez-Arroyo O., Riffo-Campos A.L., Ortega A., Forner M.J., Cortes R. (2023). Plasma Exosomal Non-Coding RNA Profile Associated with Renal Damage Reveals Potential Therapeutic Targets in Lupus Nephritis. Int. J. Mol. Sci..

[16] Cheng L., Sun X., Scicluna B.J., Coleman B.M., Hill A.F. (2014). Characterization and Deep Sequencing Analysis of Exosomal and Non-Exosomal miRNA in Human Urine. Kidney Int..

[17] Erdbrügger U., Le T.H. (2019). Extracellular Vesicles as a Novel Diagnostic and Research Tool for Patients with HTN and Kidney Disease. Am. J. Physiol. Ren. Physiol..

[18] Wang X., Gao Y., Tian N., Zou D., Shi Y., Zhang N. (2018). Astragaloside IV Improves Renal Function and Fibrosis via Inhibition of miR-21-Induced Podocyte Dedifferentiation and Mesangial Cell Activation in Diabetic Mice. Drug Des. Devel Ther..

[19] Gu Y.-Y., Liu X.-S., Huang X.-R., Yu X.-Q., Lan H.-Y. (2020). Diverse Role of TGF-β in Kidney Disease. Front. Cell Dev. Biol..

[20] Nasci V.L., Chuppa S., Griswold L., Goodreau K.A., Dash R.K., Kriegel A.J. (2019). miR-21-5p Regulates Mitochondrial Respiration and Lipid Content in H9C2 Cells. Am. J. Physiol. Heart Circ. Physiol..

[21] Zhao S., Li W., Yu W., Rao T., Li H., Ruan Y., Yuan R., Li C., Ning J., Li S. (2021). Exosomal miR-21 from Tubular Cells Contributes to Renal Fibrosis by Activating Fibroblasts via Targeting PTEN in Obstructed Kidneys. Theranostics.

[22] Larrue R., Fellah S., Van der Hauwaert C., Hennino M.-F., Perrais M., Lionet A., Glowacki F., Pottier N., Cauffiez C. (2022). The Versatile Role of miR-21 in Renal Homeostasis and Diseases. Cells.

[23] Li Z., Deng X., Kang Z., Wang Y., Xia T., Ding N., Yin Y. (2016). Elevation of miR-21, through Targeting MKK3, May Be Involved in Ischemia Pretreatment Protection from Ischemia-Reperfusion Induced Kidney Injury. J. Nephrol..

[24] Lu X., Yu Y., Tan S. (2020). The Role of the miR-21-5p-Mediated Inflammatory Pathway in Ulcerative Colitis. Exp. Ther. Med..

[25] Khalaji A., Mehrtabar S., Jabraeilipour A., Doustar N., Rahmani Youshanlouei H., Tahavvori A., Fattahi P., Alavi S.M.A., Taha S.R., Fazlollahpour-Naghibi A. (2024). Inhibitory Effect of microRNA-21 on Pathways and Mechanisms Involved in Cardiac Fibrosis Development. Ther. Adv. Cardiovasc. Dis..

[26] Zhan L., Mu Z., Jiang H., Zhang S., Pang Y., Jin H., Chen J., Jia C., Guo H. (2023). MiR-21-5p Protects against Ischemic Stroke by Targeting IL-6R. Ann. Transl. Med..

[27] Ortiz A., Lorz C., Egido J. (1999). The Fas Ligand/Fas System in Renal Injury. Nephrol. Dial. Transplant..

[28] Wang G., Yu Y., Sun C., Liu T., Liang T., Zhan L., Lin X., Feng X.-H. (2016). STAT3 Selectively Interacts with Smad3 to Antagonize TGF-β Signalling. Oncogene.

[29] Bharti N., Agrawal V., Kamthan S., Prasad N., Agarwal V. (2023). Blood TGF-Β1 and miRNA-21-5p Levels Predict Renal Fibrosis and Outcome in IgA Nephropathy. Int. Urol. Nephrol..

[30] Loboda A., Sobczak M., Jozkowicz A., Dulak J. (2016). TGF-Β1/Smads and miR-21 in Renal Fibrosis and Inflammation. Mediat. Inflamm..

[31] Wang J.-Y., Gao Y.-B., Zhang N., Zou D.-W., Wang P., Zhu Z.-Y., Li J.-Y., Zhou S.-N., Wang S.-C., Wang Y.-Y. (2014). miR-21 Overexpression Enhances TGF-Β1-Induced Epithelial-to-Mesenchymal Transition by Target Smad7 and Aggravates Renal Damage in Diabetic Nephropathy. Mol. Cell. Endocrinol..

[32] Liu L., Liu L., Liu R., Liu J., Cheng Q. (2023). Exosomal miR-21-5p Derived from Multiple Myeloma Cells Promote Renal Epithelial-Mesenchymal Transition through Targeting TGF-β/SMAD7 Signalling Pathway. Clin. Exp. Pharmacol. Physiol..

[33] Climent M., Viggiani G., Chen Y.-W., Coulis G., Castaldi A. (2020). MicroRNA and ROS Crosstalk in Cardiac and Pulmonary Diseases. Int. J. Mol. Sci..

[34] Pratheeshkumar P., Son Y.-O., Divya S.P., Turcios L., Roy R.V., Hitron J.A., Wang L., Kim D., Dai J., Asha P. (2016). Hexavalent Chromium Induces Malignant Transformation of Human Lung Bronchial Epithelial Cells via ROS-Dependent Activation of miR-21-PDCD4 Signaling. Oncotarget.

[35] Dhas Y., Arshad N., Biswas N., Jones L.D., Ashili S. (2023). MicroRNA-21 Silencing in Diabetic Nephropathy: Insights on Therapeutic Strategies. Biomedicines.

[36] Yasen A., Feng J., Xie X.-M., Li K., Cai Y.-H., Liao Z.-H., Liang R.-B., Dai T.-X., Wang G.-Y. (2023). Exosomes Derived from TGF-Β1-Pretreated Mesenchymal Stem Cells Alleviate Biliary Ischemia-Reperfusion Injury through Jagged1/Notch1/SOX9 Pathway. Int. Immunopharmacol..

[37] Li J., Wang Z.-H., Sun Y.-H. (2023). TGF-Β1 Stimulated Mesenchymal Stem Cells-Generated Exosomal miR-29a Promotes the Proliferation, Migration and Fibrogenesis of Tenocytes by Targeting FABP3. Cytokine.

[38] Ilg M.M., Bustin S.A., Ralph D.J., Cellek S. (2024). TGF-Β1 Induces Formation of TSG-6-Enriched Extracellular Vesicles in Fibroblasts Which Can Prevent Myofibroblast Transformation by Modulating Erk1/2 Phosphorylation. Sci. Rep..

[39] Zang J., Maxwell A.P., Simpson D.A., McKay G.J. (2019). Differential Expression of Urinary Exosomal MicroRNAs miR-21-5p and miR-30b-5p in Individuals with Diabetic Kidney Disease. Sci. Rep..

[40] Kidney Disease: Improving Global Outcomes (KDIGO) CKD Work Group (2024). KDIGO 2024 Clinical Practice Guideline for the Evaluation and Management of Chronic Kidney Disease. Kidney Int..

[41] Martinez-Arroyo O., Flores-Chova A., Sanchez-Garcia B., Redon J., Cortes R., Ortega A. (2023). Rab3A/Rab27A System Silencing Ameliorates High Glucose-Induced Injury in Podocytes. Biology.

