# Supplementary material for: Enrichment of RedoxifibromiR miR-21-5p in Plasma Exosomes of Hypertensive Patients with Renal Injury
Source: Int J Mol Sci. 2025 Jan 12;26(2):590. doi: 10.3390/ijms26020590 (PMC11765217; doi:10.3390/ijms26020590)
Supplement: Supplementary file 1 [file ijms-26-00590-s001.zip › ijms-3395982-supplementary.pdf]

Supplemental Table S1. Clinical characteristics of discovery cohort

Variables	DN (n = 15)	CNT (n = 25)
Age (years)	63.5 ± 9.9***	40 ± 12
Gender (male)	87%	60%
SBP (mmHg)	139 ± 25**	120 ± 10
DBP (mmHg)	82 ± 11*	75 ± 75
PP (mmHg)	58 ± 19*	45 ± 7
Glucose (mg/dL)	146 ± 56**	99 ± 12
Glycated hemoglobin (%)	7.1 ± 1.2**	5.6 ± 0.4
Total Cholesterol (mg/dL)	166 ± 26	185 ± 60
LDL (mg/dL)	96 ± 19*	117 ± 45
HDL (mg/dL)	42 ± 11**	57 ± 18
Triglycerides (mg/dL)	253 ± 220***	88 ± 54
Plasma creatinine (mg/dL)	1.27 ± 0.39***	0.75 ± 0.27
GFR (mL/min/1.73 m^2)	68 ± 25*	102 ± 20
Body mass index (kg/m^2)	34 ± 7***	24 ± 4
Obesity grade (%)		
Grade I	7	7
Grade II	47**	0
Grade III	13**	0
Diabetes (%)	100***	6
Dyslipidemia (%)	100***	6
Smoking (%)	20	13
UAE/Creatinine (mg/g)	513.9 ± 193.1***	2.8 ± 1.1
Treatment (%)		
Oral antidiabetic	80***	6
Insulin	73***	0
CCB	40**	0
ARAII	87***	13
Statins	87***	13
BB	20	0
Diuretics	67**	0

ARA II: angiotensin receptor antagonists II; BB: beta blockers; CCB: calcium channel blockers; CNT: control; DBP: diastolic blood pressure; DN: diabetic nephropathy; GFR: glomerular filtration rate; HDL: High-density lipoprotein; LDL: Low-density lipoprotein; SBP: systolic blood pressure; UAE: urinary albumin excretion. * p-value <0.05; ** p-value < 0.01; *** p-value < 0.0001.

Supplemental Table S2. Pathway enrichment analysis for miR-21-5p by WebGestalt (Gene SeT AnaLysis Toolkit).

KEGG Pathways	Enrichment ratio	Count of genes	FDR
MicroRNAs in cancer	8.2011	5	0.00072
Proteoglycans in cancer	12.402	5	0.00092
Chemokine signaling pathway	10.593	4	0.0118
Ras signaling pathway	8.6181	4	0.0182
Cytokine-cytokine receptor interaction	6.8481	4	0.0338
Pathways in cancer	4.7879	5	0.0338
Lipid and atherosclerosis	11.054	3	0.0387
Type I diabetes mellitus	23.650	2	0.0430
MAPK signaling pathway	7.0678	3	0.0430
Hepatitis C	9.6545	3	0.0430
Influenza A	8.9205	3	0.0430
Allograft rejection	26.762	2	0.0430

FDR: False discovery rate; KEGG: Kyoto Encyclopedia of Genes and Genomes; Mitogen-activated protein kinase (MAPK);
